# Epidemiological and clinical data of hospitalizations associated with respiratory syncytial virus infection in children under 5 years of age in Spain: FIVE multicenter study

**DOI:** 10.1111/irv.12224

**Published:** 2014-01-02

**Authors:** David Moreno-Perez, Cristina Calvo, on behalf of Five Study Group

**Affiliations:** a Regional Universitario HospitalMálaga, Spain; b Severo Ochoa HospitalMadrid, Spain

**Keywords:** Children, hospitalization, respiratory syncytial virus, special populations

## Abstract

**Background:**

Respiratory syncytial virus (RSV) is an important pathogen in lower respiratory tract infections (LRTI) in infants, but there are limited data concerning patients with underlying conditions and children older than 2 years of age.

**Methods:**

We have designed a prospective observational multicenter national study performed in 26 Spanish hospitals (December 2011–March 2012). Investigational cases were defined as children with underlying chronic diseases and were compared with a group of previously healthy children (proportion 1:2). Clinical data were compared between the groups.

**Results:**

A total of 1763 children hospitalized due to RSV infection during the inclusion period were analyzed. Of them, 225 cases and 460 healthy children were enrolled in the study. Underlying diseases observed were respiratory (64%), cardiovascular (25%), and neurologic (12%), as well as chromosomal abnormalities (7·5%), immunodeficiencies (6·7%), and inborn errors of metabolism (3·5%). Cases were statistically older than previously healthy children (average age: 16·3 versus 5·5 months). Cases experienced hypoxemia more frequently (*P* < 0·001), but patients with respiratory diseases required oxygen therapy more often (OR: 2·99; 95% CI: 1·03–8·65). Mechanical ventilation was used more in patients with cardiac diseases (OR: 3·0; 95% CI: 1·07–8·44) and in those with inborn errors of metabolism (OR: 12·27; 95% CI: 2·11–71·47). This subgroup showed a higher risk of admission to the PICU (OR: 6·7, 95% CI: 1·18–38·04). Diagnosis of pneumonia was more frequently found in cases (18·2% versus 9·3%; *P* < 0·01).

**Conclusions:**

A significant percentage of children with RSV infection have underlying diseases and the illness severity is higher than in healthy children.

## Introduction

Respiratory syncytial virus (RSV) is an important pathogen in lower respiratory tract infections (LRTI) in infants. By the age of 2 years, more than 90% of children have already experienced at least one RSV infection and 0·5–2% have required hospitalization.^1^–^3^ Morbidity and mortality associated with RSV infections are higher in preterm infants, especially in those with chronic pulmonary disease and in young children with hemodynamically significant congenital heart diseases. For both groups of patients, specific programs regarding prophylaxis with monoclonal antibodies have been developed.^4^,^5^ Very young infants (under 1 month of age) also need intensive care treatment more frequently during the RSV epidemic season.^6^

Respiratory syncytial virus infection is well characterized in children younger than 2 years of age, but little is known in older children. We also have limited data concerning other groups of children with underlying conditions or chronic diseases. Children with neuromuscular diseases and chronic lung diseases, for example, have been proven to have a higher risk of morbidity and mortality due to RSV infection,^7^–^9^ and Mori *et al*.^10^ have described more severe RSV disease in immunocompromised children as compared to immunocompetent ones in Japan.

We have carried out a prospective multicenter observational study in Spain with the objective of estimating the percentage of children having underlying diseases among the total number of hospitalized patients under 5 years of age with RSV infection. We compared the clinical characteristics of two groups of patients, those “with” versus “without” underlying diseases. As far as we know, this is the first national prospective study of the epidemiology of RSV infection in children under the age of 5 years in Spain.

## Patients and methods

This is a national, prospective, multicenter, study performed in 28 pediatric hospitals proportionally representative of all Spanish communities. Recorded data in two hospitals did not follow the study protocol, and finally 26 hospitals were included. The study protocol was first approved by the Ethics Committee of the Carlos Haya Hospital (Malaga) and then by each participating hospital ethics committee.

### Clinical assessment

The population studied included children younger than 5 years of age with confirmed RSV respiratory tract infection who were admitted to participating hospitals between December 2011 and March 2012. For each enrolled patient, informed consent was obtained from the parents or legal guardians. All patients were evaluated by an attending physician. We compared children with underlying chronic diseases (defined in Table 1) with children without any underlying chronic diseases. For each child enrolled with previous diseases as a case during the study period, the next two children hospitalized due to RSV infection without underlying chronic diseases were included. In order to guarantee a geographic representation, recruitment was not competitive. In each hospital, a maximum of 12 cases could be included in the study. A total of 1763 children with confirmed RSV infection were admitted to the 26 participating hospitals, and 264 children with underlying diseases were considered as investigational cases (15·0%). A total of 232 cases and 464 previously healthy children accepted to participate and were enrolled in the study. Of those enrolled, four healthy children and six cases were excluded because data were incomplete and one case because the child was older than 5 years of age. Eventually, 225 cases and 460 previously healthy children were analyzed.

**Table 1:** Case group: Underlying diseases by subgroups

Underlying diseases	N (%)
Respiratory diseases	145 (64·4)
Asthma (3 or more episodes of recurrent wheezing diagnosed by a physician)	100
Bronchopulmonary dysplasia	32
Bronchomalacia	5
Malformations of the respiratory system	5
Others (tracheostomy, diaphragmatic paralysis)	11
Cardiovascular diseases	56 (24·9)
Congenital non-cyanotic cardiac defects	43
Cyanotic congenital cardiac defects (left–right shunt)	7
Pulmonary hypertension	4
Arrhythmias	3
Myocardiopathy	2
Neurologic diseases	28 (12·4)
Cerebral palsy	19
Hypotonic syndrome	4
Refractory epilepsy	2
Myopathy	1
Myelomeningocele	1
Others (stroke, leucomalacia)	5
Chromosomal abnormalities	17 (7·6)
Down's syndrome	12
Others (Ondine syndrome, polymalformative syndromes)	5
Immunocompromised	15 (6·7)
Primary combined immunodeficiency	3
Leukemia	1
Solid tumor	4
Immunosuppressive therapy	2
Organ transplant recipient	2
Others (thalassemia major, sickle cell disease, cyclic neutropenia)	6
Inborn errors of metabolism	8 (3·6)
Congenital hypothyroidism	4
Phenylketonuria	1
Adrenal insufficiency	1
Pompe disease	1
Others (malnutrition and digestive disorders)	6 (2·7)

During the hospital stay, and as part of the study, a physician filled out a study questionnaire with the following variables: age, sex, clinical diagnosis, history of prematurity and underlying chronic diseases, requirement for oxygen therapy as determined by transcutaneous oxygen saturation, fever (axillary temperature >38°C), presence of infiltrate/atelectasis on chest radiography, administration of antibiotic therapy, fluids or enteral feeding, duration of hospital stay, intensive care admission, and non-invasive or mechanical ventilation. Clinical definitions in the protocol were as follows: upper respiratory tract infection (URTI), diagnosed in patients with rhinorrhea and/or cough, no signs of wheezing, dyspnea, rales, or bronchodilator use, with or without fever. Asthma was diagnosed on the basis of the National Asthma Education and Prevention Program guidelines. All other episodes of acute expiratory wheezing were considered to be recurrent wheezing. Acute expiratory wheezing was considered to be bronchiolitis when it occurred for the first time in children aged <2 years. *Laryngotracheobronchitis* was associated with increased effort on inspiration and wheezing, and *laryngitis* with increased effort on inspiration without wheezing. Cases with both focal infiltrates and consolidation on chest X-rays were, in the absence of wheezing, classified as *pneumonia*. Respiratory distress was considered as difficulty breathing as demonstrated in a child with accessory respiratory muscle use, tachypnea, suprasternal or subcostal retractions.

Specimens from patients were collected using nasopharyngeal aspirates and were taken from each subject upon admission. Specimens were processed immediately using an enzyme-linked immunosorbent assay.

### Statistical analysis

The estimate of the proportion of children hospitalized with RSV who had an underlying disease was measured by relative frequency (percentage) with a confidence interval of 95%. Descriptive statistics were measured by absolute frequencies and percentages for qualitative data. For quantitative data, the mean and standard deviation (SD) or median and upper and lower quartiles were used. The comparison between the two groups (i.e., those with versus without chronic underlying diseases) for qualitative variables was made using chi-square test and Fisher's exact test, with at least one expected frequency of <5. The comparison of quantitative nonparametric variables was performed using Student's *t*-test or Mann–Whitney *U*-test when two groups were compared and analysis of variance (anova) or Kruskal–Wallis test when more than two independent groups were compared. *P*-values <0·05 were considered statistically significant. Assessment of risk factors associated with more severe RSV disease was made using multivariate logistic regression for binary outcome variables and multiple linear regression models for continuous outcome variables. All results were calculated both non-adjusted and adjusted by age.

## Results

### Demographic and epidemiological data

In total, 685 patients were analyzed: 225 patients (32·9%) were included in the case group (underlying diseases) and 460 patients (67·1%) in the group of previously healthy children. The final ratio of cases versus healthy children was 2·04.

Cases were statistically significantly older (average age: 16·3 months; range: 0·3–59 months) than healthy children (5·5 months; 0·3–46 months) (*P* < 0·001) (Table 2). By underlying condition subgroup, immunocompromised patients were on average the oldest (21·2 months) and metabolic patients the youngest (5·2 months). Therefore, all results were adjusted by age.

**Table 2:** Epidemiological and clinical features

	Case group (underlying diseases)	Previously healthy group	*P* value
Median age (IQR Q1-Q3) (months)	12·49 (5·26–23·21)	2·96 (1·32–6·80)	<0·001
Prematurity (≤37 weeks)	67/155 (43·2%)	63/386 (16·3%)	<0·001
Palivizumab (previous administration)	30/179 (16·7%)	8/425 (1·8%)	<0·001
Duration of fever (mean) (days)	2·89	2·24	<0·001
Poor feeding	38/225 (16·9%)	81/460 (17%)	n.s.
Respiratory distress	182/225 (80·9%)	354/460 (77·0%)	n.s.
Hypoxemia at admission (O2 Sat <92%)	114/225 (50·7%)	151/460 (32·8%)	<0·001
Chest X-ray	172/225 (76·4%)	243/460 (52·8%)	<0·001
Pathologic chest X-ray	137/172 (79·6%)	190/243 (78·2%)	n.s.

IQR, interquartile range; Q1, quartile 1; Q3, quartile 3; n.s., non-significant.

More males were enrolled in the study than females (ratio: 1·58) both in cases (1·47) and in previously healthy children (1·64), but the difference was not statistically significant (*P* = 0·56).

A larger percentage of children with underlying diseases attended day care or school than previously healthy children (67·6% versus 14·8%, *P* < 0·001). No differences were observed between the groups when variables such as number of siblings (*P* = 0·14) and smokers in the home (*P* = 0·38) were analyzed.

The percentage of preterm infants observed in the case group (*n* = 67/225, 43·2%) was larger than in the group of previously healthy children (*n* = 58/460, 16·3%) (*P* < 0·001). In terms of weeks of gestation, preterms in the case group were younger (median: 30·8 weeks) than in the previously healthy children (median: 34·4 weeks) (*P* < 0·001).

A history of administration of palivizumab was more frequent in the case group (16·7% versus 1·8%, *P* < 0·001) (Table 2).

### Underlying conditions in case group

During the study period (from the first case to the last patient included in each center), 1763 children under 5 years of age were admitted because of RSV infection in 26 Spanish hospitals. Of them, 264 (15·0%) were children with underlying conditions and therefore potential candidates to be included in the study. This proportion ranged between 3·4% and 33·3% among centers. We calculated a weighted percentage, quantifying for the heterogeneity between centers, to be 15·2% (95% CI: 12·51–18·11%). This percentage was calculated using a random effect model.

In the case group, 225 patients were included. Of them, 183 patients (81·3%) had at least one underlying disease, 35 (15·6%) two diseases, and 7 (3·1%) three diseases.

The distribution by underlying conditions in the case group is shown in Table 1. Children with respiratory diseases formed the predominant group (145 patients, 64·4%), followed by those with cardiovascular diseases, neurologic diseases, chromosomal abnormalities, immunocompromised patients, and children with inborn errors of metabolism.

The predominant underlying respiratory disease was asthma (=3 or more episodes of recurrent wheezing diagnosed by a physician), which included 100 cases, that is, 69·0% of the total number of respiratory patients. Most of these patients suffered from an episodic form of asthma (48 cases, 48·0%).

### Clinical features on admission

The differences between cases and previously healthy children are shown in Table 2. Patients with underlying conditions experienced fever more frequently, which was higher in degree and lasted longer than the other group. Respiratory distress was similar between the groups (*P* = 0·28), but cases showed hypoxemia (oxygen saturation <92% on room air) more frequently (50·7%) than previously healthy children (32·8%) (*P* < 0·001).

Thoracic radiographs were performed more often among cases with underlying diseases (76·4% versus 52·8%; *P* < 0·001), but no differences were observed in terms of pathological findings (*P* = 0·81).

### Therapeutic measures regarded as severity markers

Some markers of hospitalization and therapeutic measures were associated with a higher degree of severity in the case group. These items are shown in Table 3. Use of an intravenous line, antibiotics, and enteral nutrition by nasogastric tube was significantly more frequent in the case group (*P* < 0·01). Antibiotic therapy was more frequently used in immunocompromised patients (OR: 4·37; 95% CI: 1·13–16·95).

**Table 3:** Severity markers of therapeutic measures in both groups

	Case group (underlying diseases)	Previously healthy group	*P* value
Oxygen therapy	184/225 (81·8%)	334/458 (72·9%)	<0·05
Median duration of oxygen therapy (IQR Q1-Q3) (days)	3·0 (1·0–5·0)	2·0 (0·0–3·0)	<0·001
High-flow oxygen therapy	32/225 (14·2%)	33/460 (7·2%)	<0·01
Median length of high-flow oxygen therapy (IQR Q1-Q3) (days)	3·5 (2·0–5·75)	2·0 (1·75–3·0)	<0·05
Non-invasive ventilation	24/225 (10·7%)	36/460 (7·8%)	n.s.
Median length of non-invasive ventilation (IQR Q1-Q3) (days)	4·0 (2·0–7·5)	2·0 (1·0–4·0)	0·057
Mechanical ventilation	14/225 (6·2%)	8/460 (1·7%)	<0·01
Median length of mechanical ventilation (IQR Q1-Q3) (days)	7·0 (4·5–13·2)	6·0 (5·7–6·0)	n.s.
Venous line	114/225 (50·7%)	160/452 (35·4%)	<0·001
Nasogastric tube feeding	41/212 (19·3%)	42/419 (10·0%)	<0·01
Antibiotic therapy	111/225 (49·3%)	136/452 (30·1%)	<0·001
Length of stay (mean, days ± SD)	8·03 (1·3)	5·35 (0·3)	<0·01
Admitted to PICU since first day of hospitalization	13/225 (5·8%)	22/460 (4·8%)	n.s.
Admitted to PICU during hospitalization	42/225 (18·6%)	52/454 (11·5%)	<0·05
Median length of stay in PICU (IQR Q1-Q3) (days)	5·0 (3·0–10·0)	4·0 (2·0–5·0)	<0·05

PICU, pediatric intensive care unit; SD, standard deviation; n.s., non-significant; IQR, interquartile range.

Patients with underlying conditions required oxygen therapy more frequently and for longer periods of time than children without underlying diseases, even when the need for high-flow oxygen therapy was analyzed independently. On the other hand, there was no association with the use of non-invasive ventilation (Table 3). The main significant markers of severity were calculated both non-adjusted and adjusted by age and are reflected in Table 4. Patients with two or more underlying diseases were at a higher risk of requiring oxygen (OR: 3·7, 95% CI: 1·08–12·7, *P* = 0·03) and fluid therapy (OR: 2·1, 95% CI; 1·08–4·4, *P* = 0·03) than children with only one comorbidity (data adjusted by age).

**Table 4:** Markers of severity in patients with underlying diseases compared with previously healthy children adjusted by age

	Raw data			Adjusted by age data		
	OR	95% CI	*P*	OR	95% CI	*P*
PICU admission	1·77	(1·14–2·76),	0·0145	2·58	(1·56–4·25)	0·0002
Fever	1·46	(1·05–2·03)	0·0292	0·9	(0·62–1·32)	0·6023
Oxygen requirement	1·67	(1·12–2·48)	0·0145	1·66	(1·06–2·58)	0·0262
Mechanical ventilation.	1·81	(1·09–3)	0·0285	3·64	(2·05–6·44)	0·0001
Venous line	1·87	(1·35–2·59)	0·0002	1·86	(1·29–2·68)	0·0009
Antibiotic treatment	2·26	(1·63–3·15)	<0·0001	1·73	(1·2–2·51)	0·0037
Nasogastric tube feeding	2·15	(1·35–3·43)	0·0017	3·26	(1·93–5·51)	0·0001
Days of hospitalization	2·68	(1·34–4·03)	0·0001	3·69	(2·55–4·83)	0·0001
Stay at PICU	4	(1·28–7·52)	0·0069	4·87	(1·8–7·94)	0·0026

OR, odds ratio; CI, confidence interval; PICU, pediatric intensive care unit.

However, when comparisons of cases versus previously healthy children were made according to subgroups of pathologies, the probability of using oxygen therapy was significantly higher for the cases only in patients with underlying respiratory diseases (OR: 2·99; 95% CI: 1·03–8·65) (Table 5).

**Table 5:** Markers of severity in patients with underlying disease subgroups compared with previously healthy children (odds ratio and confidence interval)

	Oxygen therapy	Mechanical ventilation	PICU admission
Respiratory diseases (*n* = 145)	2·99 (1·03–8·65)*(*n* = 121)	0·70 (0·26–1·87) (*n* = 13)	0·59 (0·24–1·44) (*n* = 19)
Cardiac diseases (*n* = 56)	2·94 (0·94–9·23) (*n* = 48)	3·00 (1·07–8·44)** (*n* = 13)	2·08 (0·82–5·30) (*n* = 16)
Neurologic diseases (*n* = 28)	2·34 (0·65–8·47) (*n* = 24)	2·56 (0·86–7·60) (*n* = 6)	1·70 (0·62–4·67) (*n* = 7)
Chromosomal defects (*n* = 17)	2·87 (0·54–15·36) (*n* = 15)	1·03 (0·26–4·12) (*n* = 3)	0·66 (0·17–2·58) (*n* = 3)
Immunocompromised (*n* = 15)	1·33 (0·33–5·41) (*n* = 12)	2·77 (0·71–10·72) (*n* = 4)	2·08 (0·60–7·25) (*n* = 5)
Inborn errors of metabolism (*n* = 8)	3·02 (0·16–54·72) (*n* = 6)	12·27 (2·11–71·47)*** (*n* = 3)	**6·70 (1·18–38·04)**† (*n* = 3)

PICU, pediatric intensive care unit.

Results in boldface are statistically significant.

* *P* < 0·05,

** *P* < 0·05,

*** *P* < 0·01,

† *P* < 0·05.

Mechanical ventilation was used in a significantly greater proportion of patients with cardiac diseases (OR: 3·0; 95% CI: 1·07–8·44) and in those with inborn errors of metabolism (OR: 12·27; 95% CI: 2·11–71·47). Patients with inborn errors of metabolism showed a higher risk of admission to the pediatric intensive care unit (PICU) than the other subgroups (OR: 6·7, 95% CI: 1·18–38·04) (Table 5).

An additional subanalysis of the group of patients with a history of recurrent wheezing was performed and adjusted by age. This group of children required oxygen therapy (OR: 1·77, 95% CI: 1·02–3·07, *P* = 0·04), nasogastric tube feeding (OR: 2·99, 95% CI: 1·49–5·99, *P* = 0·002), and mechanical ventilation (OR: 2·68, 95% CI: 1·22–5·88, *P* = 0·01) more frequently than previously healthy children. Nevertheless, when we compared the other patients with underlying diseases to the recurrent wheezing group, PICU admission (OR: 2·5, 95% CI: 1·26–5·02, *P* = 0·009), mechanical ventilation (OR: 2·38, 95% CI: 1·06–5·31, *P* = 0·03), and nasogastric tube feeding (OR: 2·39, 95% CI: 1·18–4·83, *P* = 0·01) were more frequent in patients with other underlying diseases.

### Final diagnosis and outcome

A final diagnosis of pneumonia was more frequent in cases than in previously healthy children (18·2% versus 9·3%; *P* < 0·01). Other complications observed, all in the case group, were pleural effusion (3/225; 1·3%) and pneumothorax 1/225 (0·8%).

There were two deaths (2/225, 0·9%), both in the case group. One patient was a 4-month-old infant with severe combined immunodeficiency and hepatoblastoma with metastasis. The other one was a 2-year 8-month-old child with a terminal neuroblastoma. Both of them developed severe respiratory distress syndrome.

## Discussion

We present a large prospective, multicenter study of children under 5 years of age hospitalized due to RSV infection in Spain. This is the first epidemiological study conducted in our country in this population. The main objective of the study was to identify and characterize risk groups or children with underlying diseases who are hospitalized for RSV infection in our community. Our data show that approximately 15% of hospitalized children have underlying diseases, especially respiratory, cardiac, and neurologic diseases, but other groups, such as immunocompromised children or those with chromosomal and metabolic diseases, also represent a significant percentage. Children with underlying diseases have certain markers of disease severity at admission (Table 2). During hospitalization, they are admitted for longer periods of time, have a higher requirement for oxygen therapy, and also more often require hospitalization in intensive care units. They show a higher percentage of complications than children without any underlying disease.

This is a comparative study between children with underlying diseases and previously healthy children under 5 years of age. The characteristics of the previously healthy group show that our population is consistent with the epidemiology of RSV infection widely known and published in normally healthy children without underlying disease. The average age was 5·5 months (median: 3 months), and the diagnosis was bronchiolitis in 86% as is typically the case.^11^–^16^ Pneumonia only represented 9·6% of the cases. A history of prematurity (average gestational age of 34 weeks) was seen in 16·3%, but only eight children (1·8%) had received prophylaxis with palivizumab. Fifty-two children (11·5%) were admitted to the PICU. Although young age and incidence of prematurity could have raised the degree of severity in this group, children with underlying diseases had a worse outcome in most of the parameters analyzed when the results were adjusted by age, with a regression model.

The proportion of children with underlying diseases has been analyzed in other series. However, all studies found were retrospective. Kristensen *et al*.,^17^ in a population cohort study in Denmark found that 8·8% of all children under 2 years of age hospitalized with RSV infection had a chronic condition. They found that chronic diseases such as immunodeficiency, cerebral palsy, and other neuromuscular diseases, hepatic disease, inborn errors of metabolism, cystic fibrosis, chromosomal defects, and malformation syndromes incurred a higher risk of RSV hospitalization (*P* < 0·01). Our study shows a higher percentage of patients with underlying diseases (15%) most likely because we included children with a diagnosis of asthma (more than three episodes of wheezing diagnosed by a physician). Children with respiratory diseases comprised the most prevalent group in our series. Moreover, the distribution of diseases in our series is similar to the data found in the Danish cohort. Patients with inborn errors of metabolism also showed high risk, as in the group of Kristensen *et al*.^17^ These authors speculate that the higher rate of admission for this last group was explained by gastric intolerance; however, this does not appear to be the case in our series because our hospitalized children were at high risk of admission at PICU and mechanical ventilation risk. We have no explanation for these results. Other groups, such as cardiac and neurologic patients or immunodeficient children, were similar in our cohort.

In a retrospective study conducted in Japan during two consecutive RSV epidemic seasons, Mori *et al*.^10^ found in a nationwide survey that 1115 children under 4 years of age who did not meet the criteria for palivizumab therapy were hospitalized for severe RSV infection. Children with pre-existing diseases (*n* = 756) were compared with healthy controls (*n* = 359). Respiratory diseases represented the main group in the series (54·8%), mainly asthma (46·0%), followed by chromosomal abnormalities and malformation syndromes (17·2%; 7·5% with Down's syndrome without cardiopathy) and neuromuscular disorders (16·5%). Another similarity with our series was the higher median age in patients with chronic diseases (20·4 versus 6·7 months). Sixteen patients (1·4%) died, eleven with underlying diseases, mainly with chromosomal defects, neuromuscular diseases, or immunocompromised. A risk of poor outcome from RSV disease was approximately 1000 times higher in immunocompromised children than in those with respiratory disorders and approximately 2·8–4·3 times higher than in patients with other underlying conditions. They concluded that palivizumab should be considered in other patients with underlying conditions at risk of severe RSV infection in whom prevention of RSV infection by standard control measures appears to be difficult.

In a cohort study of children with severe RSV infection conducted in England between 1999 and 2007, all the children who died of RSV infection (*n* = 35) had underlying conditions (relative risk: 2·36). Multiple pre-existing diseases (RR: 4·38) and congenital cardiac defects (RR: 2·98) were considered risk factors for death from severe RSV infection.^8^

Recently, additional data from England have been reported. Pockett *et al*.^18^ found that children under 5 years of age with chronic diseases such as cystic fibrosis, insulin-dependent diabetes mellitus, cancer, or epilepsy hospitalized with RSV or rotavirus infection in comparison with healthy controls were older (1·1 years, SD: 1·3 years), had greater length of hospital stay (9·9 days, SD: 19·9), and incurred a higher cost (£3477, SD: £7765) than healthy controls (age: 0·2 years, SD: 0·5, *P* < 0·001; length of stay 1·9 days, SD: 3·1, *P* < 0·001; cost £595, SD: £727, *P* < 0·001). Cost for cases was six times higher than in healthy controls (*P* < 0·001).

We found that 12·4% of our cases was represented by a group of patients with neurologic diseases. In a prospective multicentric German study (1999–2005), patients hospitalized with RSV infection and neuromuscular disease showed a greater risk of requiring mechanical ventilation (9·6% versus 1·9%), with a statistically significant higher degree of mortality compared to controls (5·5% versus 0·2%).^7^

In a prospective Spanish study, Down's syndrome was identified as an independent risk factor of RSV hospitalization (relative risk: 2·6; 95% CI: 1·4–4·7).^19^ In a study conducted in Colorado (USA), children with Down's syndrome were at a higher risk of being hospitalized with RSV LRTI even in the absence of coexisting risk factors (OR: 3·5; 95% CI: 3·10–4·12).^20^

We have recognized two main potential limitations in our study. First, this study was conducted in a single epidemiological season; therefore, the variability between seasons could not be determined, either with higher or lower severity of infections occurring between years. However, the results for healthy children are absolutely comparable to studies conducted in our area during several epidemiological seasons.^11^ The second observed limitation is that previously healthy children had an average age significantly lower than the case group, in such a way that the two groups may not be comparable. However, healthy children hospitalized because of RSV infection tended to be young, and the severity was even higher in the youngest children, giving more value to data regarding severity that we found in the case group, where children were older. And the most important parameters of severity were significantly worse in children with underlying diseases when raw data were adjusted by age.

In summary, we know that a significant percentage of children with RSV infection have underlying diseases. The duration of hospitalization and the severity of the illness were both higher in these patients than in healthy children. In our series, patients with respiratory diseases had an increased risk of oxygen therapy requirement; children with heart diseases had a higher risk of requiring mechanical ventilation, and children with inborn errors of metabolism had a very high risk of admission to PICU and requiring mechanical ventilation. Awareness and strategies that decrease the risk and burden of RSV infections in these groups of children may be beneficial in addressing the respiratory health among this vulnerable population. The potential role of palivizumab in some special populations that do not meet current criteria of the guidelines for the prevention of severe RSV infection is a present subject of debate. Larger prospective studies are needed to identify underlying conditions with higher risk of severe RSV infection.^21^

## Appendix 1

### Members of the FIVE Study Group

S. Alfayate Miguélez (H Virgen Arrixaca. Murcia), C. Alvarez Alvarez (H. Marqués de Valdecilla. Santander), F. Álvez González (H. Santiago Compostela), J.V. Arcos Machancoses (H. La Fé. Valencia), J. Arístegui Fernández (H. Universitario Basurto), F. Baquero Artigao (H. Infantil La Paz. Madrid), E. Bernaola Iturbe (H. Virgen del Camino. Pamplona), S. Bueno Pardo (H. Central de Asturias. Oviedo), MJ. Cabero Pérez (H. Marqués de Valdecilla. Santander), G. Cabrera Roca (H. Materno de las Palmas), C. Calvo (H. Universitario Severo Ochoa. Leganés. Madrid.), I.M. Ceballos Rodríguez (H. Materno Infantil Badajoz), S. Cerdán Oncala (H. Albacete), L.M. Ciria Calavia (H. Miguel Servet. Zaragoza), J.A. Couceiro Gianzo (H. Pontevedra), J.M. de Cea (H. Universitario Severo Ochoa. Leganés. Madrid.), F. de Juan Martín (H. Miguel Servet. Zaragoza), M. Delgado Cardoso (H. Badajoz. Badajoz), G.M. Escudero Bueno (H. Salamanca), M. Fernández de Sevilla (H. San Joan de Deu. Barcelona), L. Fernández Silveira (H. 12 de Octubre. Madrid), M. García Barreiro (H. Pontevedra), M.J. García de Miguel (H. Infantil La Paz. Madrid), M.L. García-García (H. Universitario Severo Ochoa. Leganés. Madrid), F. Giménez Sánchez (H. Torrecárdenas. Almería), A. Gimeno Díaz de Atauri (H. Puerta de Hierro-Majadahonda. Madrid), M. González González (H. Clínico Universitario de Salamanca), M.I. González Sánchez (H. Gregorio Marañón. Madrid), M.I. González Tomé (H. Universitario 12 de Octubre. Madrid), B. González García (H. Rio Hortega. Valladolid), S. Guillén Martín (H. Getafe. Madrid), X. Hernández Fernández (H. Universitario Basurto), J.F. Hurtado Díaz (H. Pontevedra), M. Lillo Lillo (H. Albacete), M. López Sousa (Santiago de Compostela), S. Martínez Megías (H. Materno-Infantil de Las Palmas), A.I. Menasalvas Ruiz (H. Virgen Arrixaca. Murcia), D. Moreno-Pérez (H. Regional Universitario. Málaga), C. Otero Reigada (H. La Fé. Valencia), M.J. Peláez Cantero (H. Regional Universitario. Málaga), R. Pérez Gorricho (H. Niño Jesús. Madrid), M.E. Pérez Gutiérrez (H. Rio Hortega. Valladolid), R. Piñeiro Pérez (H. Puerta de Hierro-Majadahonda. Madrid), R. Rodríguez Fernández (H. Universitario Gregorio Marañón. Madrid), Ruiz del Arbol Sánchez P. (H. Central de Asturias), M. Ruiz Jiménez (H. de Getafe. Madrid), J.M. Rumbao Aguirre (H. Reina Sofía. Córdoba), M. Sánchez Forte (H. Torrecárdenas. Almería), M.A. Tejero Hernández (H Reina Sofía. Córdoba), M. Tobeña Rué (H. Vall de Hebrón. Barcelona), M. Triviño Rodríguez (H. Sant Joan de Deu. Barcelona), A. Urda Cardona (H. Regional Universitario. Málaga), N. Viguria Sánchez (H. Virgen del Camino. Pamplona).
